# ADP-Dependent Kinases From the Archaeal Order *Methanosarcinales* Adapt to Salt by a Non-canonical Evolutionarily Conserved Strategy

**DOI:** 10.3389/fmicb.2018.01305

**Published:** 2018-06-26

**Authors:** Felipe Gonzalez-Ordenes, Pablo A. Cea, Nicolás Fuentes-Ugarte, Sebastián M. Muñoz, Ricardo A. Zamora, Diego Leonardo, Richard C. Garratt, Victor Castro-Fernandez, Victoria Guixé

**Affiliations:** ^1^Laboratorio de Bioquímica y Biología Molecular, Departamento de Biología, Facultad de Ciencias, Universidad de Chile, Santiago, Chile; ^2^São Carlos Institute of Physics, University of São Paulo at São Carlos, São Paulo, Brazil

**Keywords:** *Methanosarcinales*, halophiles, ancestral protein reconstruction, halophilic adaptation, osmolytes, ADP-dependent kinases

## Abstract

Halophilic organisms inhabit hypersaline environments where the extreme ionic conditions and osmotic pressure have driven the evolution of molecular adaptation mechanisms. Understanding such mechanisms is limited by the common difficulties encountered in cultivating such organisms. Within the *Euryarchaeota*, for example, only the *Halobacteria* and the order *Methanosarcinales* include readily cultivable halophilic species. Furthermore, only the former have been extensively studied in terms of their component proteins. Here, in order to redress this imbalance, we investigate the halophilic adaptation of glycolytic enzymes from the ADP-dependent phosphofructokinase/glucokinase family (ADP-PFK/GK) derived from organisms of the order *Methanosarcinales.* Structural analysis of proteins from non-halophilic and halophilic *Methanosarcinales* shows an almost identical composition and distribution of amino acids on both the surface and within the core. However, these differ from those observed in *Halobacteria* or *Eukarya.* Proteins from *Methanosarcinales* display a remarkable increase in surface lysine content and have no reduction to the hydrophobic core, contrary to the features ubiquitously observed in *Halobacteria* and which are thought to be the main features responsible for their halophilic properties. Biochemical characterization of recombinant ADP-PFK/GK from *M. evestigatum* (halophilic) and *M. mazei* (non-halophilic) shows the activity of both these extant enzymes to be only moderately inhibited by salt. Nonetheless, its activity over time is notoriously stabilized by salt. Furthermore, glycine betaine has a protective effect against KCl inhibition and enhances the thermal stability of both enzymes. The resurrection of the last common ancestor of ADP-PFK/GK from *Methanosarcinales* shows that the ancestral enzyme displays an extremely high salt tolerance and thermal stability. Structure determination of the ancestral protein reveals unique traits such as an increase in the Lys and Glu content at the protein surface and yet no reduction to the volume of the hydrophobic core. Our results suggest that the halophilic character is an ancient trait in the evolution of this protein family and that proteins from *Methanosarcinales* have adapted to highly saline environments by a non-canonical strategy, different from that currently proposed for *Halobacteria*. These results open up new avenues for the search and development of novel salt tolerant biocatalysts.

## Introduction

Halophilic organisms are those inhabiting challenging osmotic environments, whose salinity ranges from moderate to extreme, where salts reach the limit of their solubility. These organisms are present in the three domains of life, clustering into well-defined phylogenetic groups (Oren, 2008; Weinisch et al., 2018). In archaea, halophilic organisms have been identified in the phylum *Euryarchaeota* where they are present in three phylogenetic groups; *Nanohaloarchaea* (as yet uncultivated group of organisms) (Narasingarao et al., 2012), *Halobacteria* and *Methanosarcinales* (Oren, 2008). Although organisms from *Halobacteria* and *Methanosarcinales* show different phenotypes, they have been recognized to be phylogenetically related (Burggraf et al., 1991). *Halobacteria* is the largest phylogenetic class in *Euryarchaeota* and, due to the extreme salinity required for its growth, has become the classical model for halophilic adaptation studies (Larsen, 1967; DasSarma and DasSarma, 2015). With few exceptions, these aerobic organisms are not able to grow at salinities below 3 M NaCl and thrive at its limits (5 M NaCl). Since this extreme environment imposes a large osmotic pressure, *Halobacteria* have developed a counterbalance mechanism to survive via the accumulation of up to 4 M K^+^ and Cl^-^ ions in their cytosol (Christian and Waltho, 1962; Youssef et al., 2014). Evolutionary pressure has also driven the acquisition of unique modifications to the molecular machinery of these organisms. For example, the existence of a biased amino acid composition in proteins from *Halobacteria* (Reistad, 1970; Lanyi, 1974) has been recognized for more than 50 years. These modifications rely on the interplay of the following strategies: (i) an increase in the content of acidic residues (Asp and Glu) along with a decrease in the content of Lys, leading to a negatively charged molecular surface, which constitutes the most prominent feature of proteins from *Halobacteria*, (ii) the decrease in Lys also leads to a reduction in the solvent exposed hydrophobic area, and (iii) a decrease in the overall content of large hydrophobic residues (Ile, Leu, Phe, Met) and a concomitant preference for smaller residues such as Ala and Val leading to a reduction in the volume of the hydrophobic core (Paul et al., 2008; Graziano and Merlino, 2014; Nath, 2016).

Although the order *Methanosarcinales* includes halophilic organisms, the strategies responsible for protein adaptation to high salt concentrations in these organisms have yet to be addressed. In terms of their salt requirements, these anaerobic organisms are more versatile than *Halobacteria*, since they grow in environments with salinities ranging from 0 to 5 M NaCl and therefore also include non-halophilic organisms. In order to adjust their osmotic pressure to different salinities, organisms from *Methanosarcinales* exploit a mixed osmotic counterbalance mechanism based on the accumulation of inorganic ions (reaching up to 3 M K^+^ in most halophilic organisms) (Lai and Gunsalus, 1992) and the intracellular accumulation of several small organic molecules called osmolytes known as the “compatible solutes” strategy (Lai et al., 1991). Among the osmolytes used, glycine betaine is one of those preferentially accumulated by organisms inhabiting the saltiest environments (Lai et al., 1991).

Until now there have been no reports regarding the mechanism of halophilic adaptation encountered in proteins from *Methanosarcinales*. Given their close phylogenetic relationship with *Halobacteria*, it would be interesting to ask if both groups share a common strategy or if proteins from the *Methanosarcinales* have evolved a different one. On the other hand, since the order *Methanosarcinales* includes both halophilic and non-halophilic organisms it would also be interesting to determine if the halophilic trait is ancestral or if it represents an evolutionary novelty.

To address these questions we adopted the ADP-dependent sugar kinases from *Methanosarcinales* as a model system. These enzymes belong to the ribokinase superfamily and catalyze the phosphorylation of glucose or fructose 6-phosphate employing ADP as the phosphoryl donor in glycolysis, a central pathway for energy production. Some of the superfamily members are substrate specific for glucose or fructose-6P (GK-ADP or PFK-ADP) while others are bifunctional enzymes (PFK/GK-ADP). Recently, it was reported that the enzymes from Methasanosarcinales, which have been annotated to be ADP-specific, are in fact bifunctional and therefore able to catalyze the phosphorylation of both substrates (Zamora et al., 2017). By combined bioinformatics and experimental approaches along with ancestral enzyme reconstruction, we determined the structural properties that determine the halophilic character in these proteins and the evolutionarily trajectory of this trait.

## Materials and Methods

### Protein Homology Models and Structural Analyses

Sequences for homology modeling of ADP-dependent sugar kinases from *Halobacteria, Methanosarcinales* and *Eukarya* were retrieved from UniProt and NCBI databases (Supplementary Table S1). Models built were divided into four groups based on the taxonomic categorization of the source organism and their ability to grow in high salinity environments as reported in the literature. The groups defined were: *Halobacteria*, halophilic *Methanosarcinales*, non-halophilic *Methanosarcinales*, and *Eukarya*, the latter used as a control outgroup. Six sequences were employed in each group, except for halophilic *Methanosarcinales*, which has only five sequences available. Homology models for archaeal enzymes were built employing four template structures; the ADP-GK from *Thermococcus litoralis* (PDB: 4B8R), the ADP-GK and ADP-PFK from *Pyrococcus horikoshii* (PDB: 1L2L and 1U2X, respectively), and the last common ancestor of *Methanococcales* and *Thermococcales* (PDB: 5K27). For *Eukaryotic* enzymes two templates were employed; the ADP-GK from *T. litoralis* and the ADP-GK from *Mus musculus* (PDB: 5CCF). Since eukaryotic proteins have an insertion forming an N-terminal helical domain (Richter et al., 2016), only the homologous region that contains the catalytic domain was modeled. Fifty models for each protein were generated using Modeller v9.15 (Eswar et al., 2008) and evaluated based on the DOPE potential. Upon visual inspection of the best models, those presenting abnormal loop configurations were refined using the GalaxyWEB server (Ko et al., 2012). The quality of the resulting models was assessed with ProSA-web (Wiederstein and Sippl, 2007), Procheck (Laskowski et al., 1993), and Verify3D (Eisenberg et al., 1997). The best models generated were subjected to energy minimization using a standard protocol in NAMD with the CHARMM27 force field for further analyses (Phillips et al., 2005).

To evaluate the residue distribution over the different structural models, the molecular surface area of each residue was determined using the WHAT-IF webserver (Vriend, 1990). Residues were separated into two categories based on their accessible surface area; if this was ≥5 Å^2^, the residue was considered to be surface exposed (outer shell) (Supplementary Table S2), otherwise, it was taken to be buried within the protein core (inner shell) (Supplementary Table S3). The existence of statistically significant differences was assessed by the Kruskal-Wallis test coupled to Dunn’s *post hoc* test. Polar and charged accessible surface area was determined using the VADAR web server (Willard et al., 2003). The electrostatic potential surfaces of representative models were generated using the APBS Tools plugin for PyMOL.

### Inference of Phylogeny and Ancestral Sequence Reconstruction

The ancestral sequence for the last common ancestor of the *Methanosarcinales* enzymes was inferred using the Hierarchical Bayesian method (Huelsenbeck and Bollback, 2001), employing CpREV as the evolutionary model. An alignment of all the sequences from the ADP-dependent family (120 sequences in January 2015) and an updated inferred phylogenetic tree is shown in Supplementary Figure S1. The analysis was performed with 10^7^ generations and a sampling frequency of 1000. From the resulting 20,000 trees, 40% were discarded in the burn-in step and finally 12,000 trees were employed in the calculation of the posterior probabilities. For each position of the alignment, the residue with the highest posterior probability (PP) was selected. The resulting sequence was corrected for the presence of gaps according to the method of Hall (Hall, 2006), yielding a final sequence of 490 amino acid residues. This sequence was inspected for the presence of conserved motifs and catalytic residues, all of which were present with a posterior probability equal to 1.0. The distribution of probabilities for the inferred ancestral sequence is summarized in Supplementary Figure S2.

### Protein Expression and Purification

ADP-dependent phosphofructokinase (PFK) from *Methanohalobium evestigatum* (MevePFK/GK) was purified as described previously (Zamora et al., 2017). PFK from *Methanosarcina mazei* (MmazPFK/GK) was purified as follows: *Escherichia coli* C41 cells were transformed with the plasmid pET-15b harboring the *de novo* and codon-optimized gene for the MmazPFK/GK protein sequence (UNIPROT code: Q8PZL9), including a poly-histidine-tag at the N-terminus and ampicillin resistance. An overnight culture of these cells was employed to inoculate 4 L of terrific broth containing 100 μg mL^-1^ ampicillin. Protein expression was induced at an OD of 0.6 with 0.5 mM IPTG. Cells were grown overnight at 30°C and then the medium was centrifuged at 8,000 *g* for 10 min to obtain a cell pellet which was suspended in lysis buffer (1 M NaCl, 25 mM Tris pH 7,8, 5 mM MgCl_2_, 1 mM PMSF) and disrupted by sonication at 4°C. This volume was centrifuged at 15000 *g* for 20 min to remove cell debris and the resulting supernatant was heated at 50°C for 30 min. This volume was again centrifuged at 15,000 *g* for 20 min to remove denatured protein and the resulting supernatant was loaded onto a 5 mL Ni^+2^-NTA column (GE-Healthcare). The protein was eluted with a linear gradient of imidazole from 20 to 300 mM (25 mM Tris pH 7.8 and 500 mM NaCl). Fractions with ADP-dependent kinase activity were pooled and dialyzed against 1 L of buffer (20 mM Tris pH 8.2, 100 mM NaCl). The dialyzed protein was loaded onto a HiTrap-Q anion exchange column (GE-Healthcare) and eluted with a linear gradient from 100 to 1000 mM NaCl. Fractions with enzyme activity were pooled and dialyzed against buffer (25 mM Tris pH 8.2, 300 mM NaCl, and 5 mM MgCl_2_).

For the last common ancestor of PFKs from *Methanosarcinales* (AncMsPFK/GK) the gene sequence was codon-optimized for expression in *E. coli* and was synthesized by GENSCRIPT (Piscataway, NJ, United States), then cloned into pET-15. BL21 *E. coli* cells were transformed with the expression vector and cultivated in 1L of LB medium with 100 μg mL^-1^ ampicillin until reaching an OD_600_ of 0.6. The protein expression was induced using 1 mM IPTG overnight at 37°C. Cells were centrifuged (8,000 *g* for 15 min at 4°C) and suspended in lysis buffer (25 mM Tris HCl pH 7.8, 20 mM imidazole, 1 M NaCl and 5 mM MgCl_2_). Subsequently, cells were disrupted by sonication (12 pulses of 20 s, with 40% amplitude and 1 min intervals) and the resulting lysate was centrifuged (15,000 *g* for 20 min at 4°C). The supernatant was heated at 80°C for 20 min and the denatured proteins were removed by centrifugation (15,000 *g* for 20 min at 4°C). The supernatant was filtrated and loaded onto a High Performance Ni^+2^-Sepharose 5 mL column (GE Healthcare) equilibrated with Buffer A (25 mM Tris HCl pH 7.8, 20 mM imidazole, 500 mM NaCl, and 5 mM MgCl_2_). The protein was eluted using a linear gradient of 20 to 200 mM imidazole in the same buffer. The active fractions were pooled and dialyzed against protein storage buffer (25 mM Tris HCl pH 7.8, 300 mM NaCl, 5 mM MgCl_2_, and 50% glycerol) and stored at -80°C.

### Enzyme Activity Measurements

PFK and GK ADP-dependent activities were determined as previously reported (Kengen et al., 1994). Briefly, the GK activity was determined by following the reduction of NAD^+^ at 340 nm in a coupled assay with 10–13 units of G6PDH from *Leuconostoc mesenteroides*, which was heterologously expressed in *E. coli* and purified in our laboratory. The assay also contained 25 mM HEPES pH 7.8 and 0.5 mM NAD^+^. The PFK-ADP activity was measured by following the oxidation of NADH at 340 nm in a coupled assay with the following auxiliary enzymes: α-glycerol-3-phosphate dehydrogenase (5 units), triosephosphate isomerase (50 units) and aldolase (1.3 units) all from rabbit muscle (Sigma-Aldrich), along with 25 mM PIPES pH 6.5 and 0.2 mM NADH. All enzyme activity determinations were performed at 40°C and the specific activity was calculated from the initial velocity data. The unit of enzyme activity (U) was defined as the conversion of 1 μmol of substrate per minute. Kinetic parameters for the PFK and GK-ADP activities were determined varying the concentration of one substrate at a fixed and saturating concentration of the co-substrate. The initial velocities determined were adjusted either to the Michaelis-Menten or substrate inhibition equations by non-linear regression.

### Glucokinase Activity Determinations in the Presence of Salt

GK-ADP activity was chosen to monitor the effects of NaCl and KCl on the activity of MevePFK/GK and MmazPFK/GK, because the auxiliary enzyme used in the continuous GK-ADP assay is not inhibited by high concentrations of these salts. For both enzymes, the reaction mixture contained saturating concentrations of the substrates glucose and Mg-ADP (according to the kinetic characterization of each enzyme), 25 mM HEPES pH 7.8, 0.5 mM NAD^+^ and 40 units/mL of G6PDH.

### Phosphofructokinase Activity Determinations in the Presence of Salt

To assess the effect of salts on the AncMsPFK/GK PFK activity a discontinuous assay was employed, since the auxiliary enzymes of the continuous PFK-ADP assay are inhibited by NaCl concentrations above 300 mM. The enzyme activity was determined at 40°C in an incubation mixture containing 25 mM Pipes pH 6.5, 15 mM MgCl_2_, 3 mM fructose-6P, 10 mM ADP, and 0.004 U of AncMsPFK/GK in a final volume of 0.20 mL. The reaction was stopped by the addition of perchloric acid to a final concentration of 3.7% v/v. The solution was cooled on ice for 5 min and neutralized by adding sodium bicarbonate. Subsequently the sample was centrifuged at 18,000 *g* and 4°C for 10 min and the soluble fraction recovered. The product of the PFK-ADP reaction, fructose 1,6-bisphosphate, was quantified by spectrophotometric titration to the endpoint by following NADH oxidation at 340 nm. Essentially, the titration reaction mixture contained 50 mM Pipes pH 6.5, 0.2 mM NADH, 5 U of α-glycerophosphate dehydrogenase, 50 U of triose phosphate isomerase and 1.3 U of aldolase in a final volume of 0.50 mL. The effect of KCl on the AncMsPFK/GK PFK activity was not assayed due to the interference of this salt with the quenching reaction.

### Thermal Stability

Protein thermal stability was analyzed by following the secondary structure content in a Jasco spectropolarimeter J-1500, equipped with a peltier system (PTC-517), using a cuvette of 1 mm path length. Protein concentrations from 2 to 5 μM were employed and the samples were prepared in 20 mM sodium phosphate buffer pH 7.8 at different KCl or glycine betaine concentrations. The change in CD signal was monitored at 222 nm with a temperature ramp of 1°C/min.

### Kinetic Stability Assays

Proteins were incubated in 20 mM HEPES buffer pH 7.0 and different KCl concentrations (0, 0.1, 1, and 2 M) at a final protein concentration ranging from 0.02 to 0.03 mg mL^-1^. MevePFK/GK/GK and MmazPFK/GK/GK solutions were incubated at 40°C, but given its thermophilic character the AncMsPFK/GK assay was performed at 60°C. At the times indicated in **Figure 6**, samples were taken and centrifuged (13,000 × *g* for 20 min) to remove denatured protein. For the three enzymes the PFK-ADP activity was monitored on a microplate reader at 30°C (Sinergy 2, Biotek) employing a 250 μL reaction volume and saturating substrate concentrations.

### X-Ray Crystallography

Purified MeveFK/GK, MmazPFK/GK and ancMsPFK/GK were initially screened for crystallization conditions with commercial kits using an ARI Gryphon crystallization robot (Arts Robbins Instruments LLC) by the sitting-drop vapor-diffusion method. Preliminary crystals were only obtained for ancMsPFK/GK using the PEGrx reagent kit (Hampton Research Corp) leading to subsequent rounds of optimization. Good quality crystals were obtained using a drop of 1 μL of protein (10 mg mL^-1^, 2.5 mM ADP, 200 mM NaCl, 7.5 mM MgCl_2_, 25 mM HEPES, pH 7.8, and 1 mM 2-mercaptoethanol) and 1 μL of the reservoir solution containing 20 mM CdCl_2_, 20 mM MgCl_2_, 20 mM NiCl_2_, 24% PEG MME 2000 and 100 mM sodium acetate, and pH 4.5. Crystals appeared after 2 weeks at 19°C and were soaked in a cryoprotective solution consisting of the crystallization solution plus 25% PEG 400, and flash-frozen in liquid nitrogen. X-ray diffraction data were collected at 100K on the MX2 beamline at the Brazilian Synchrotron Light Laboratory (LNLS Campinas-SP) using a PILATUS 2M detector (Dectris Ltd.) and an X-ray wavelength of 1.459 Å. The data were indexed, integrated, and scaled with XDS (Kabsch, 2010), and merged using Aimless from the CCP4 package (Evans and Murshudov, 2013). The phases were solved by molecular replacement with Molrep (Vagin and Teplyakov, 2010), using the structure of the ancestral ADP-dependent kinase (PDB ID 5K27; Castro-Fernandez et al., 2017) split into small and large domains as search models. The structure was refined using Phenix (Adams et al., 2010) and COOT (Emsley and Cowtan, 2004). The ADP, water, magnesium, and nickel ions were placed using COOT and refined with ion restraints in Phenix. Full data collection and refinement statistics are summarized in **Table 2**.

## Results

### Phylogenic Analysis of *Methanosarcinales* Enzymes

**FIGURE 1:**
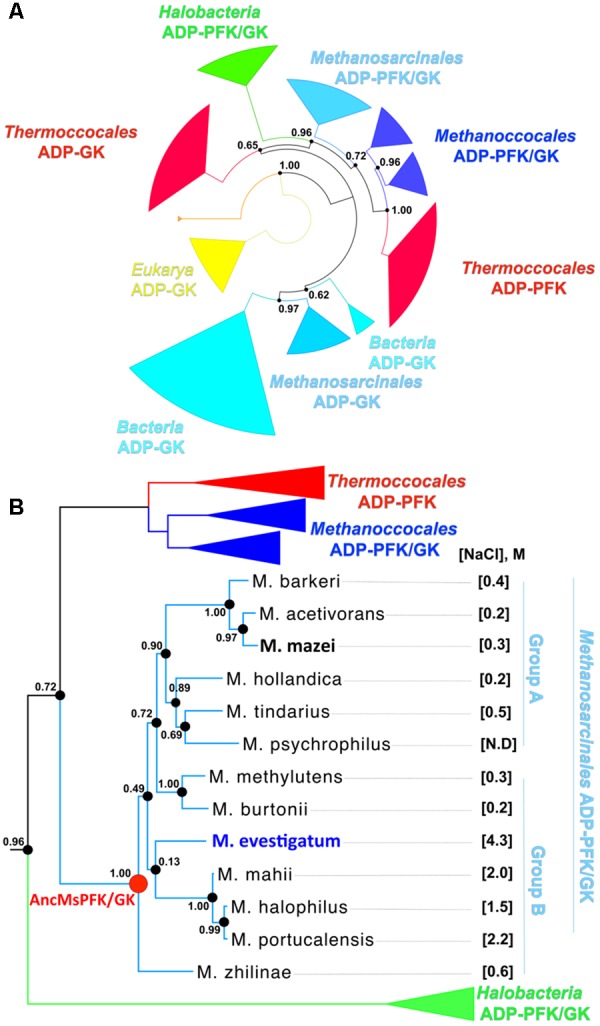
Phylogenetic relationships among *Euryarchaeota* ADP-dependent kinases. **(A)** Maximum Likelihood phylogenetic tree for ADP-dependent kinases from sequenced organisms. Colors represent different phylogenetic groups. The likelihood ratio test values of each node are indicated. **(B)** Diagram of the *Methanosarcinales* ADP-dependent kinase family. The parentheses indicate the optimum NaCl molar concentration of growth for each organism. N.D. stands for “not determined”. The most halophilic organism of the group (*M. evestigatum*) is highlighted in blue, the non-halophilic organism (*M. mazei*) in black and the last common ancestor of the group (ancMs) in red. *M. zhilinae* is able to grow up to 2.5 M of NaCl, therefore it was included in halophilic organisms.

In order to characterize the distribution of the ADP-PFK/GK enzymes present in the order *Methanosarcinales*, we retrieved the deposited sequences from the non-redundant database and updated a previous phylogenetic tree reported for the same protein family (Zamora et al., 2017). As shown in **Figure 1**, ADP-PFK/GKs are present in all organisms from *Methanosarcinales* sequenced to date (13 in total), and encompasses species inhabiting environments ranging from 0 to 5 M NaCl (Supplementary Table S1).

The inferred tree (**Figure 1** and Supplementary Figure S1) shows that *Halobacteria* and *Methanosarcinales* are closely related phylogenetic groups. The topology of the *Methanosarcinales* group shows that most of the ADP-PFK/GKs from non-halophilic organisms cluster together in group A. However, two (from *Methanococcoides burtonni* and *Methanococcoides methylutens*) are phylogenetically more closely related to those from halophilic organisms (*Methanohalobium evestigatum, Methanohalophilus mahii, Methanosalsum zhilinae, Methanohalophilus halophilus and Methanohalophilus portucalensis*) and comprise group B. The presence of these two groups is consistent with previous phylogenies reported for *Methanosarcinales* organisms (Liu, 2010). Given the phylogenetic proximity of *Halobacteria* and *Methanosarcinales* and the presence of halophilic organisms in both groups, an interesting question is if all such proteins are adapted to high salt concentrations by a common strategy. Furthermore, given that the order *Methanosarcinales* includes both halophilic and non-halophilic organisms, we evaluated if the halophilic character is a conserved or an emergent trait during evolution.

### Amino Acid Composition Analyses

**FIGURE 2:**
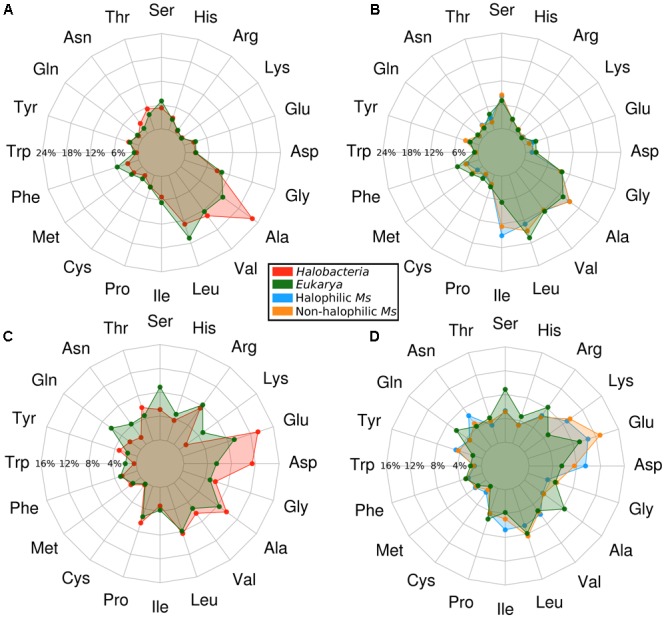
Amino acid composition of the inner and outer shell of the ADP-dependent kinases from *Halobacteria* and *Methanosarcinales.* Points on the graphs represent the average percentage of each residue located in the respective shell of the homology models. All the results are compared against ADP-dependent kinases from *Eukarya* as a control. **(A)** Inner shell of proteins from *Halobacteria*. **(B)** Inner shell of proteins from *Methanosarcinales* (Ms). **(C)** Outer shell proteins from *Halobacteria*. **(D)** Outer shell of proteins from *Methanosarcinales* (Ms).

To assess the structural adaptations present in the enzymes from *Methanosarcinales*, we built homology models and evaluated the residue composition and distribution for proteins from halophilic and non-halophilic organisms. These data were compared with models generated for enzymes from both *Halobacteria* and *Eukarya* which served as controls for extremely halophilic and non-halophilic proteins, respectively. The average amino acid content is shown in **Figure 2** (Supplementary Tables S2, S3). In each graph, the results for *Halobacteria* and both halophilic and non-halophilic *Methanosarcinales* proteins are always compared to the results obtained for proteins from *Eukarya*. ADP-PFKs from *Halobacteria* display the classical amino acid distribution reported for proteins from this phylogenetic class (Fukuchi et al., 2003; Paul et al., 2008). The inner shell of *Halobacteria* proteins is characterized by an increase in the small residue Ala (70% compared to *Eukarya*) and by a decrease in large hydrophobic residues such as Ile, Met, Leu, Phe, and Trp (22, 20, 20, 49, and 69%, respectively) (**Figure 2A**). Interestingly, there are no significant differences between the inner shells of proteins from halophilic and non-halophilic *Methanosarcinales* organisms (**Figure 2B**). Nevertheless, when this distribution is compared to that observed for the inner shell of proteins from *Eukarya*, neither of the *Methanosarcinales* groups show an increase in the content of small hydrophobic residues but rather, a significant increase in the content of the large hydrophobic residue Ile (97% for halophilic Ms and 93% for non-halophilic Ms). Notably, this amino acid composition differs from that described for *Halobacteria*, where an increase in content of small hydrophobic residues has been pointed out to be a key component of the adaptation mechanism (Paul et al., 2008; Nath, 2016). On the other hand, the outer shell of *Halobacteria* proteins is characterized by the well-known increase in acidic residues (Asp and Glu, 109 and 46%, respectively), together with a significant decrease in Lys (around 72%) and an overall reduction in neutral polar residues such as Ser, Gln, and Asn (42, 63, and 66%, respectively) (**Figure 2C**). Once again, when the outer shell of non-halophilic and halophilic *Methanosarcinales* proteins was compared, an almost identical amino acidic composition was found (**Figure 2D**). However, a noticeable increase was observed in the Asp content with halophilic proteins presenting a 72% of difference compared to *Eukarya* whereas in non-halophilic proteins this was only 39%.

The most striking difference compared to the outer shell observed in proteins from *Halobacteria* is a large increase in Lys (approximately twofold compared to *Eukarya* and sevenfold compared to *Halobacteria*). This is worth noting since some authors have suggested the Lys over Asp/Glu content to be the main basis for the halophilic character of proteins from *Halobacteria* due to a decrease in the solvent exposed hydrophobic area (Paul et al., 2008; Tadeo et al., 2009).

**FIGURE 3:**
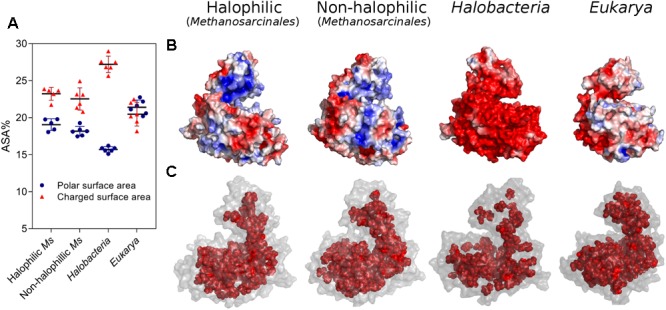
Halophilic traits of homology models constructed for ADP-PFK/GK from *Halobacteria, Eukarya*, halophilic and non-halophilic *Methanosarcinales*. **(A)** Polar and charged surface area expressed as percentage of ASA. **(B)** Electrostatic potential surface of representative models were generated, being blue for positive and red for negative charge (±3 k_B_*T*/*e*). **(C)** Residues of the protein core (inner shell), using as criteria a surface residue exposition less than 5 Å^2^. A representative model of each group is shown; *Natronorubrum bangense* (*Halobacteria*), *Homo sapiens* (*Eukarya*), *Methanohalobium evestigatum* (halophilic *Methanosarcinales*), and *Methanosarcina mazei* (non-halophilic *Methanosarcinales*).

To further analyze the residue distributions observed in the models, we assessed the two main structural features associated with salt stability that are directly correlated with the amino acid composition of a protein; the electrostatic surface charge and the hydrophobic interactions in the core. **Figure 3A** shows the polar and charged accessible surface area (ASA) for proteins from each group. Proteins from *Halobacteria* show a remarkable increase in charged ASA over polar ASA, while proteins from halophilic and non-halophilic *Methanosarcinales* organisms also present this trait but less pronounced. On the other hand, eukaryotic proteins show an almost equal amount of charged and polar ASA. These characteristics are emphasized in **Figure 3B**, which shows a representative electrostatic potential surface for each group. ADP-PFKs from *Halobacteria* have a predominantly negatively charged surface which is significantly diminished in *Eukarya*. On the other hand, both *Methanosarcinales* groups show a strong positive component on their surface probably be due to the high Lys content. **Figure 3C** shows a graphical illustration of the spatial distribution of the residues that compose the protein core. In proteins from *Halobacteria* the core is less extensive when compared to the core of proteins from eukaryotes or species of *Methanosarcinales*.

Our results show that proteins from halophilic organisms from the order *Methanosarcinales* do not share the structural traits nor the amino acid composition classically observed in *Halobacteria*, thus raising the question of how these proteins are able to carry out catalysis in molar concentrations of salt. We therefore proceeded to assess experimentally the halophilic character of the *Methanosarcinales* proteins.

### Effect of Salt and Glycine Betaine on the Activity of Extant and Ancestral *Methanosarcinales* Enzymes

The halophilic character of enzymes from *Methanosarcinales* was evaluated by choosing proteins from non-halophilic and halophilic organisms (**Figure 1B** and Supplementary Table S1). The PFK/GKs from *M. mazei* (MmazPFK/GK) and *M. evestigatum* (MevePFK/GK) were selected as representative examples, respectively. To complement the comparative study and to trace the evolution of the halophilic trait, we inferred and experimentally resurrected the last common PFK/GK-ADP ancestor of the *Methanosarcinales* order (AncMsPFK/GK) (Supplementary Figure S2) (see Section “Materials and Methods”).

**Table 1** shows that the three heterogeneously expressed enzymes are able to catalyze the phosphorylation of both substrates. This is consistent with annotations which imply that they are bifunctional enzymes. In terms of their PFK-ADP activity the three enzymes possess similar k_cat_ and K_M_ values. For their GK-ADP activity, however, differences were found between the ancestor and extant enzymes, and both MevePFK/GK and MmazPFK/GK showed similar K_M_ values for glucose while for the ancestor this was 10-fold higher. Furthermore, for the ancestral enzyme, no saturation for the ADP substrate was attained even at 30 mM, prohibiting the determination of k_cat_.

**Table 1:** Kinetic parameters of *Methanosarcinales* ADP-dependent kinases.

	MevePFK/GK^∗^	MmazPFK/GK	AncMsPFK/GK
**PFK activity**			
K_M_F6P (μM)	34 ± 3	6.6 ± 0.5	6 ± 1
K_M_MgADP (μM)	250 ± 50	72 ± 9	700 ± 100
K_i_MgADP (mM)	3.0 ± 0.8	6.1 ± 0.9	6 ± 2
k_cat_ F6P (s^-1^)	9.5 ± 0.2	4.60 ± 0.06	6.00 ± 0.09
k_cat_MgADP (s^-1^)	16 ± 2	6.1 ± 0.3	8.7 ± 0.6
k_cat_/K_M_F6P (M^-1^S^-1^)	2.79 × 10^5^	6.94 × 10^5^	9.21 × 10^5^
k_cat_/K_M_MgADP (M^-1^S^-1^)	6.27 × 10^4^	8.45 × 10^4^	1.22 × 10^4^
**GK activity**			
K_M_Glc (mM)	10.9 ± 0.8	12 ± 1	134 ± 11
K_M_MgADP (mM)	1.50 ± 0.08	0.6 ± 0.1	—
k_cat_Glc (s^-1^)	1.9 ± 0.1	1.40 ± 0.03	3.60 ± 0.09
k_cat_MgADP (s^-1^)	2.0 ± 0.1	1.7 ± 0.8	—
k_cat_/K_M_Glc (M^-1^S^-1^)	1.78 × 10^2^	1.19 × 10^2^	5.48
k_cat_/K_M_MgADP (M^-1^S^-1^)	1.3 × 10^3^	2.90 × 10^2^	—

*Measurements determined at 40°C. (—) Enzyme activity was assayed up to 30 mM but no saturation was attained. ^∗^Parameters taken from Zamora et al. (2017).*

**FIGURE 4:**
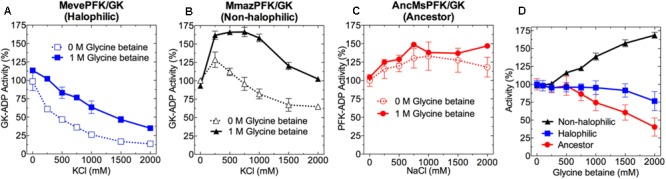
Effect of KCl, NaCl and glycine betaine on the activity of ADP-PFK/GK from *Methanosarcinales*. Effect of KCl or NaCl on the: **(A)** MevePFK/GK (halophilic), **(B)** MmazPFK/GK (non-halophilic), and **(C)** ancMsPFK/GK (ancestor) activity in the absence and presence of 1 M glycine betaine. **(D)** Activity of: MevePFK/GK, MmazPFK/GK and ancMsPFK/GK as a function of glycine betaine concentration in the absence of salt. In all cases, activity determinations were made at saturating substrate concentrations. Activity was expressed as the percentage of that obtained in the absence of both salt and glycine betaine.

At saturating substrate concentration (**Figures 4A–C** and Supplementary Figure S3) we found that extant *Methanosarcinales* enzymes behave similarly as a function of salt concentration, irrespective of whether they come from non-halophilic or halophilic organisms. Furthermore, the activity of both enzymes appears to be diminished at increased salt concentrations and, unexpectedly, the non-halophilic model presented an optimal salt requirement higher than that of the halophilic model. Considering their ability to retain significant activity at high salt concentration their behavior can be considered to be halotolerant, being similar to that observed for several enzymes from *Halobacteria* (Pugh et al., 1970; Brown-peterson and Salin, 1993; Bonete et al., 1996). Also, the effect of NaCl on the activity of MevePFK/GK and MmazPFK/GK is similar to that observed for KCl (Supplementary Figure S3). Interestingly, the ancestral enzyme is not only active at high NaCl concentrations, but it seems to be actually activated by it. However, the activity of the ancestral enzyme could not be measured in the presence of KCl, since high concentrations of this salt causes precipitation in the presence of perchloric acid.

Another relevant aspect of halophilic adaptation is the presence of glycine betaine or other osmolytes as protective agents against the harmful effects of hyperosmotic environments. When the effect of glycine betaine (1.0 M) was assayed together with molar concentrations of KCl, an apparently protective effect was observed for the MevePFK/GK and MmazPFK/GK activity, while no evident effect on the AncMsPFK/GK activity was detected. However, variable glycine betaine concentrations had very different effects on the enzymatic activity depending upon the enzyme studied. Whilst relatively little effect was observed in the case of the halophilic model, a 50% activation was seen in the case of the non-halophilic enzyme. In contrast, the ancestral enzyme was inhibited by glycine betaine, retaining only 50% of its activity at this concentration (**Figure 4D**).

### Effect of Salt and Glycine Betaine on the Stability of *Methanosarcinales* Proteins

**FIGURE 5:**
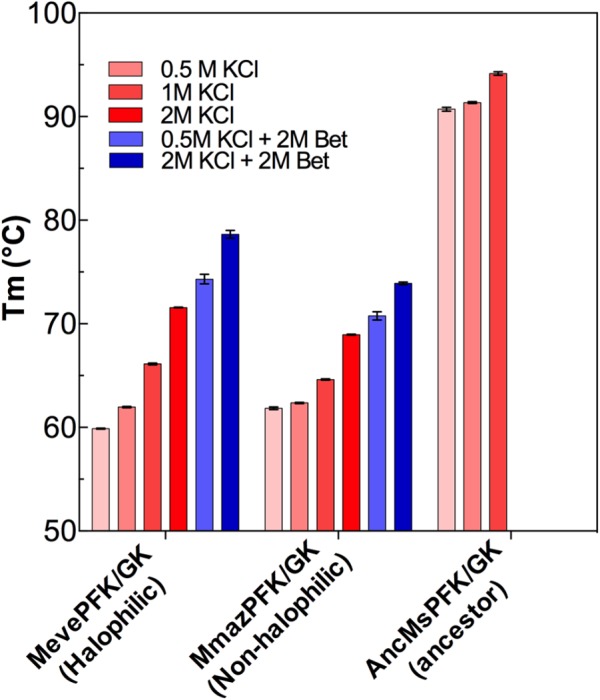
Thermal stability of ADP-dependent kinase proteins from *Methanosarcinales.* Melting temperature (Tm) as a function of KCl and glycine betaine concentrations Red bars indicate different KCl concentrations whereas blue bars depict determinations made also in the presence of glycine betaine.

Considering that *Methanosarcinales* ADP-dependent kinases are only moderately resistant to KCl and NaCl inhibition and to get a good understanding of the halophilic adaptation of these proteins, we assessed the effect of these salts on protein stability (Supplementary Figure S4). Using circular dichroism we determined the melting temperature (Tm) for both the extant *Methanosarcinales* enzymes and the ancestor in the presence of different KCl concentrations (**Figure 5**). The three enzymes displayed a linear increase in their Tm with increasing KCl concentrations, in agreement with previous studies that show the ability of chosmotropic ions to increase the melting temperature of proteins (Von Hippel and Schleich, 1969). The Tm of the halophilic MevePFK/GK protein shows the greatest increase (around 12°C) in the presence of 2.0 M salt. Noticeably, at 0.5 M KCl the Tm for the ancestral protein is approximately 30°C greater than those obtained for the extant enzymes, implying that it should be classified as a hyper-thermophilic protein. Glycine betaine produces a displacement of the Tm of MmazPFK/GK and MevePFK/GK to higher values, both at low and high KCl concentrations. In the case of MevePFK/GK and MmazPFK/GK, a partially additive effect between glycine betaine and KCl was observed, which suggests a similar mechanism underlying the increase in Tm cause by these two effectors (Roesser and Müller, 2001).

**FIGURE 6:**
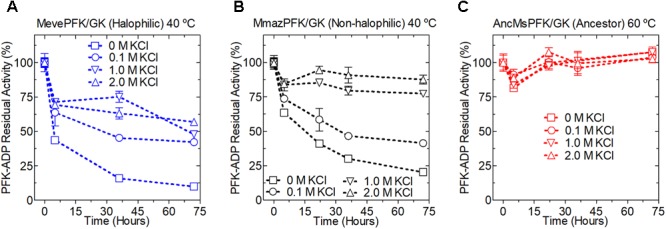
Kinetic stability of extant and ancestral ADP-dependent kinases from *Methanosarcinales*. PFK-ADP residual activity for *Methanosarcinales* enzymes were determined over time at different KCl concentrations; kinetic stability of MevePFK **(A)** and MmazPFK **(B)** was assayed at 40°C while for ancMsPFK **(C)** 60°C was employed. At the indicated times an aliquot was removed, centrifuged and its activity assayed at 30°C.

The MmazPFK/GK and MevePFK/GK proteins were unstable at low KCl concentrations and are progressively stabilized at higher concentrations. Interestingly, AncMsPFK/GK displayed a highly stable profile at all the KCl concentrations tested (0 to 2 M KCl) when incubated at 60°C (**Figure 6**). This high stability is in agreement with its hyper-thermophilic character and may be related to the increased stability shown by resurrected ancestral proteins in general (Trudeau et al., 2016).

### Structure Determination of the Last Common Ancestor of PFK/GK From *Methanosarcinales*

**FIGURE 7:**
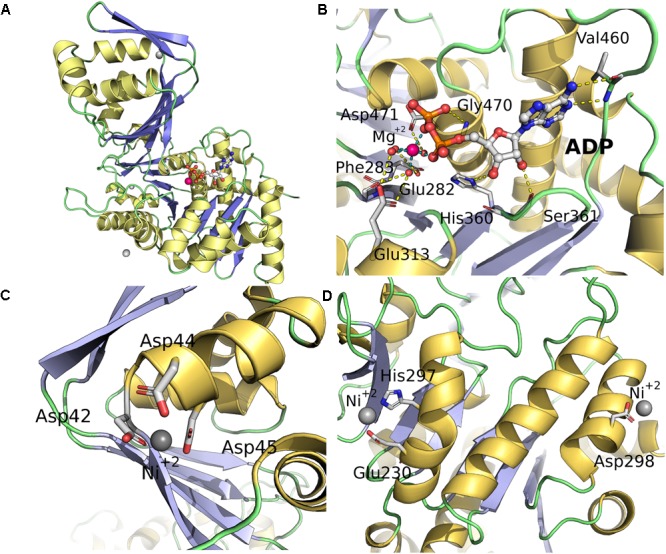
Crystallographic structure of ancMsPFK/GK, the Mg-ADP and cation binding sites. **(A)** Cartoon representation colored according to secondary structure; α-helices are colored in yellow, β-strands in blue and loops in green. The ADP molecule is shown as sticks. The Mg^2+^ and Ni^2+^ ions are shown as red and gray spheres, respectively. **(B)** ADP binding site, yellow dotted lines indicate polar contacts calculated with PyMOL. **(C)** Binding of Ni^2+^ in the minor domain coordinated by three Asp residues (42, 44, and 45). **(D)** Binding of other two Ni^2+^ ions in the major domain.

Given the absence of experimentally determined structures for ADP-dependent kinases from *Methanosarcinales*, crystallization screens for the enzymes characterized here were performed. We only obtained crystals for the ancMsPFK/GK in complex with ADP, which was solved by molecular replacement and refined to 2.86 Å resolution. Data collection and refinement statistics are given in **Table 2**. The structure of the ancestral enzyme can be divided into large and small alpha/beta domains showing the classical fold of an ADP-dependent kinase. The large domain harbors a Rossman fold (α/β/α) architecture, with eleven β-sheet strands surrounded by fourteen α-helices, and the small domain consists of seven β-strands and four α-helices, which acts as a lid for the active site (**Figure 7A**). The ADP molecule is located in a shallow pocket within the large domain where the interactions observed in other ADP-dependent kinases are conserved. (**Figure 7B**; Ito et al., 2001). The Mg^+2^ ion coordinates the α and β-phosphates of the ADP together with four water molecules (Supplementary Figure S5), revealing it to be the first structure of an ADP-dependent kinase where the active site metal interacts only indirectly with the protein through water molecules. These interactions include: the side chains of Glu313 (motif NXXE), Glu282 (motif HXE), and Thr284 and the main chain carbonyl of Phe283. The glutamic acids of the NXXE and HXE motifs have been previously described as essential for ADP-Mg binding in the protein family as a whole and for ternary complex formation at the Ribokinase superfamily level (Abarca-Lagunas et al., 2015).

**Table 2:** Data collection and refinement statistics for the ancestor structure.

	ancMsPFK/GK
**Data collection**	
X-ray source	LNLS MX2
Detector	PILATUS 2M
Wavelength (Å)	1.459
Resolution Range (Å)	37.99–2.859 (2.961–2.859)
Space group	C 1 2 1
Cell dimensions (Å) *a b c*–(°) α β γ	81.081 75.986 82.442–90.00 94.92 90.00
I/σ(I)	10.06 (1.78)
Rmerge	0.1307 (1.094)
CC1/2	0.996 (0.656)
Rpim	0.05537 (0.4625)
Completeness (%)	99.71 (99.74)
Multiplicity	6.6 (6.5)
Wilson B factor	73.93
Reflections	76687 (7501)
Unique reflections	11662 (1156)
**Refinement**	
R_work_/R_free_	0.1980/0.2386
**Number of atoms**	
Protein	3864
Ligands	31
***B*-factors**	
Protein	68.84
Ligands	68.05
Water	68.77
Coordinate error (ML based) (Å)	0.46
Phase error (°)	26.65
**R.m.s. deviations**	
Bond lengths (Å)	0.010
Bond angles (°)	1.24
**Ramachandran plot**	
Favored (%)	97.53
Allowed (%)	2.26
Outliers (%)	0.21
All atom clashscore	8.71
PDB ID	6C8Z

*Statistics for the highest-resolution shell are shown in parentheses.*

Another feature of this structure is the binding of 3 cations (Ni^2+^) which could be due to the high concentration of the metal present in the crystallization solution. One of these is located at the beginning of α-helix 2 and involves Asp42, Asp44, and Asp45 (site I) all residues coming from the same chain (**Figure 7C**). The other two sites are formed by residues making crystal-packing contacts; site II is composed of His227 and Glu230 from two symmetrically related molecules, while site III is formed by Asp298 from one molecule and Asp47 from a symmetrically related chain (**Figure 7D**).

**FIGURE 8:**
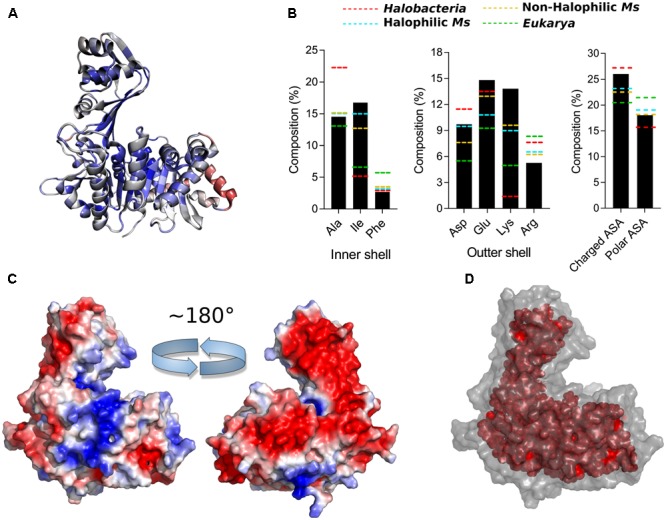
Halophilic traits of the ancestor structure. **(A)** Structure of the ancestor colored according to structural conservation through ADP-dependent kinases family using a BLOSUM30 matrix, sequence conservation from high (blue) to low (red). **(B)** Amino acid composition of main changes identified in **Figure 2** at the inner and outer shell and percentage of polar and charged exposed surface area (ASA) calculated using the VADAR web server. The respective average content for *Eukarya* (green), halophilic Ms (light blue), non-halophilic (orange) and *Halobacteria* (red) proteins is shown as dotted lines. **(C)** Electrostatic potential surface of the ancestor generated using the APBS Tools plugin for PyMOL, being blue for positive and red for negative charge (±3 k_B_*T*/*e*). The figure shows the structure rotated in 180°. **(D)** Residues of the protein core (inner shell), using as criteria a surface residue exposition less than 5 Å^2^.

Comparison of the structures of ancMsPFK/GK and the extant PFK from *Pyrococcus horikoshii* (PhPFK, PDB 1U2X) from the order *Thermococcales*, shows a very high structural conservation in the ancestral enzyme. Structural superposition of both enzymes showed an overall RMSD of 2.2 Å between α-carbons, although they share only 40% sequence identity. The ancestral enzyme is in a slightly more open conformation than PhPFK, which is evident when the distance between the centers of mass of the small and large domains are compared, being 32 Å apart in PhPFK and 34 Å in ancMsPFK/GK. Analysis of the small domain of PhPFK and ancMsPFK/GK showed exactly the same secondary structure elements with an α-carbon RMSD of 1.44 Å. However, in the large domain the insertion of some additional structural elements is observed. For example, α-helix 17 and β-strand 17 are absent in PhPFK while in the ancestral enzyme a larger helix 1 (20 residues longer) is present. Consequently, the α-carbon RMSD for the large domains is higher at 1.77 Å. Noticeably, the regions corresponding to the insertions of additional secondary structure elements observed in ancMsPFK/GK are also present in the sequences of all *Methanosarcinales* enzymes, showing that this is a structural difference present in *Methanosarcinales* ADP-PFK/GKs compared to ADP-PFKs from *Thermococcales* (**Figure 8A**).

Analysis of the key residues present in the outer shell of the ancMsPFK/GK structure shows several particular features (**Figure 8B**). In agreement with what was found in *Halobacteria* proteins, the ancestor presents a significant increase in the Glu content, even more than that observed in *Halobacteria*, together with an increase in the Asp content as well. However, most noticeable is the Lys content of the ancestor which even greater than that observed in the extant proteins from *Methanosarcinales* and reaching an almost ninefold increase when compared to proteins from *Halobacteria*.

These changes impact the polar and charged ASA of the protein (**Figure 8B**), where the size of the charged surface is similar to that found in proteins from *Halobacteria*, while the size of its polar surface remains closer to that from *Methanosarcinales* proteins. The remarkable increase in the Lys content leads to noticeable positive charge to the molecular surface as can be readily seen from the electrostatic potential of the crystal structure (**Figure 8C**). On the other hand, the inner shell of the ancMsPFK/GK protein displays a similar trend to that observed in *Methanosarcinales* proteins (**Figure 8B**), showing no preponderance of Ala residues but rather an increase in the Ile content, which in turn produced an extensive hydrophobic core (**Figure 8D**). These results indicate that the ancMsPFK/GK achieves its high salt concentration stability mainly by a highly charged surface with no reduction of its hydrophobic core.

## Discussion

Halophilic adaptations of proteins have been a matter of intense research in recent years. Nonetheless, a comprehensive model that integrates how these molecules remain soluble and face the variety of environments that halophilic organisms inhabit, is still lacking. During the last 50 years, studies have been focused on proteins from the order *Halobacteria*, which is the classical model for the study of these molecular adaptations (Lanyi, 1974). However, currently it is recognized that many halophilic organisms belong to the order *Methanosarcinales*, which shows great diversity ranging from non-halophilic species to extreme halophiles. However, the halophilic adaptations encountered in proteins from these organisms have yet to be addressed.

Three major differences in the amino acid distribution of halophilic (*Halobacteria*) and non-halophilic proteins are widely recognized. One of these is an excess of acidic residues on the protein surface, another is a significant reduction in the exposed hydrophobic area (mainly caused by a reduction in surface-exposed lysine residues) and the third is a reduction in the volume of the hydrophobic core. When comparing the amino acid composition of the inner and outer shell of ADP-dependent enzymes from halophilic and non-halophilic species of *Methanosarcinales* no significant differences were found, although they both differ from the classical signatures present in halophilic proteins from *Halobacteria* or non-halophilic proteins from *Eukarya*. In particular, there was an increase in the isoleucine content of the inner shell. This trend is opposite to that described for *Halobacteria*, where a decrease in large hydrophobic residues (Leu, Phe, Ile) has been reported (Lanyi, 1974; Paul et al., 2008; Siglioccolo et al., 2011). Conceivably the limited conformational freedom and size of the isoleucine sidechain leads to a smaller conformational entropy loss on protein folding, whilst simultaneously retaining a large hydrophobic contact area, leading to an overall gain in stability. The other known mechanism involves an increase in the alanine content which would have a greater entropic advantage but lead to a reduction in the size of the hydrophobic core. This would also be favored by the high ionic strength environment that enhances the hydrophobic effect. However, a reduction in the size of the hydrophobic core is clearly not the adaptive mechanism which has arisen in *Methanosarcinales.*

On the other hand, the outer shell of both *Methanosarcinales* groups shared with *Halobacteria* proteins an increase in Asp and Glu and a decrease in Ser. However, the most striking difference is the Lys content in the outer shell. In proteins from *Halobacteria* this residue diminishes to almost none whereas in *Methanosarcinales* proteins it increases up to twofold, when compared to *Eukarya*. This particular amino acid distribution of *Methanosarcinales* proteins supports the idea of novel and non-canonical molecular adaptations to face life in halophilic environments. Moreover, this strategy is present in *Methanosarcinales* proteins ranging all the way from non-halophilic organisms up to extreme halophiles. Clearly they have evolved a completely different strategy in terms of their hydrophobic core and yet a partially similar one in terms of their molecular surface, at least in as much as they have highly charged surfaces compared with *Eukarya*. The net increase of charged residues (Lys, Asp, Glu) in the outer shell of *Methanosarcinales* proteins may be related to the fact that these residues have been pointed out to be the best candidates for increasing protein solubility at high salt concentrations (Trevino et al., 2007). Regarding the adaptation mechanism exhibited in *Halobacteria*, the Lys content has been highlighted as the main residue responsible, since a twofold decrease in its content leads to a fourfold decrease in the hydrophobic solvent exposed area contributed by this residue (Britton et al., 2006). This fact is directly related to the destabilization exhibited by *Halobacteria* at low KCl concentrations (Tadeo et al., 2009). Taken together our results support a mechanism for adaptation to halophilic environments that differs from the current models proposed for proteins from *Halobacteria* (Madern et al., 2000; Qvist et al., 2012).

To further address the contribution of these residues to the halophilic character of the *Methanosarcinales* proteins a comprehensive mutational analysis should be performed. However, these kinds of studies are highly complex since as the pioneering mutational work on proteins from *Halobacteria* demonstrated, the same residue located at different positions at the surface of the protein may contribute differently to the halophilic character. This may be due to its potential epistatic interactions, its contribution to secondary structure formation or protein stability, among others factors (Jolley et al., 1997; Esclapez et al., 2007; Tadeo et al., 2009).

Another remarkable feature of halophilic proteins is the presence of salt as a prerequisite for activity (Mevarech et al., 2000). In many cases, a loss of enzymatic activity has been detected upon salt removal and this can be correlated with structural data. Nonetheless, some exceptions are reported where enzymes remained fully active and structured even in the absence of salt (Madern and Zaccai, 2004; Hutcheon et al., 2005). These results differ from those observed for enzymes from non-halophilic organisms which are only marginally active at NaCl/KCl concentrations higher than 0.5 M and completely inactive at molar salt concentrations (Wright et al., 2002). Thus, regarding the salt dependence of the enzymes studied here from *Methanosarcinales* species, they can be classified as moderately halophilic proteins due to the substantial preservation of enzyme activity at molar salt concentrations.

On the other hand, *Methanosarcinales* organisms present a more complex strategy towards osmotic counterbalance since they accumulate, not only intracellular inorganic ions to concentrations above 1 M, but also osmolytes like glycine betaine (Lai and Gunsalus, 1992). This osmolyte is able to protect enzyme activity against NaCl/KCl inhibition and also to increase the thermal stability of proteins (Bowlus and Somero, 1979; Pollard and Wyn Jones, 1979). Our analyses showed that the presence of 1 M glycine betaine along with high salt concentrations has a differential effect depending on the enzyme studied; it protects the enzyme activity of the halophilic and non-halophilic model enzymes but has no significant effect on the ancestor. Although further experiments are needed to validate this trend, the results suggest that glycine betaine protection of enzyme activity in the halophilic model could be an emergent trait that was absent in the last common PFK/GK-ADP ancestor. Moreover, the similar behavior observed for the *Methanosarcinales* enzymes in terms of activity and stability in the presence of salt can be explained by their similar amino acid composition, whereas the glycine betaine protective effect on the enzyme activity of the halophilic model may be related to its interaction with specific amino acids residues associated with the active site.

The halophilic character is also reflected in the circular dichroism data that show a linear increase in stability (melting temperature) as a function of the KCl concentration. Although the increase in Tm by inorganic ions is a common characteristic of proteins (Von Hippel and Schleich, 1969), this feature is more pronounced in halophilic proteins, most probably through the destabilization of the unfolded state (Ortega et al., 2015). Here, this feature was replicated for both extant and ancestral enzymes, as shown in the thermal and kinetic stability data. Consistent with previous reports describing the effect of glycine betaine on the Tm, we found that this osmolyte increases the melting temperature of *Methanosarcinales* proteins (Bowlus and Somero, 1979). However, when glycine betaine is assayed in the presence of KCl, a non-additive effect was observed suggesting that a similar mechanism may be underlying the effect of both compounds. The osmolyte mediated increase in Tm has been explained by the balance of two effects; on the one hand the ability of osmolytes to preferentially hydrate proteins leading to protein stabilization and on the other a destabilizing effect due to the modestly favorable interaction of exposed hydrophobic side chains with apolar parts of the osmolyte (Santoro et al., 1992; Auton et al., 2011). The adaptation strategies adopted by *Methanosarcinales* proteins may be the result of the co-evolution of osmolytes, which has lowered the adaptive pressure acting on its proteins.

Surprisingly, ADP-PFK/GKs from non-halophilic and halophilic organisms shared several of the halophilic traits described above. This led us to address the halophilic character of the last common ancestor of these organisms. The ancestral enzyme was as resistant to salt as halophilic proteins from *Halobacteria*. Although structurally, the ancestral protein displays no significant differences with extant ADP-dependent kinases in terms of its amino acid composition it presents several striking features. Among these, the Lys content on its surface and the lack of a reduction to the size of the hydrophobic core stand out. These features are common to those which we observe in extant enzymes and seem to be the ones that contribute most to the non-canonical strategy for halophilic adaptation in this protein family. Probably, the halophilic traits present in the ancestral enzyme have been conserved during evolution even in non-halophilic organisms as they present insufficient disadvantage to have been rapidly eliminated by natural selection and have remained as a vestige of evolution.

Strikingly, the ancestral enzyme also displays an extremely high melting temperature (90°C approximately), in agreement with previous reports showing the high stability of resurrected ancestral proteins. Furthermore, the ancestral enzyme presents high kinetic stability and elevated activity at molar salt concentrations. We argue that ancestral enzyme reconstruction opens up new lines of research to explore the halophilic character of ancestral enzymes as a potential tool in biotechnology and to unveil the molecular evolution of halophilic organisms and their proteins.

## Author Contributions

VC-F, FG-O, and VG conceived the project and designed the research PC built the protein structure models and performed the bioinformatics analyzes. FG-O, RZ, and PC performed the phylogenetic analysis. FG-O, NF-U, and SM performed the enzyme kinetic and stability experiment. FG-O, NF-U, SM, DL, RG, and VC-F performed and analyzed the protein crystallography experiment. FG-O, PC, RG, VC-F, and VG wrote the paper.

## Conflict of Interest Statement

The authors declare that the research was conducted in the absence of any commercial or financial relationships that could be construed as a potential conflict of interest.
