# Supplementary material for: ADP-Dependent Kinases From the Archaeal Order Methanosarcinales Adapt to Salt by a Non-canonical Evolutionarily Conserved Strategy
Source: Front Microbiol. 2018 Jun 26;9:1305. doi: 10.3389/fmicb.2018.01305 (PMC6028617; doi:10.3389/fmicb.2018.01305)
Supplement: Supplementary file 1 [file Table_1.PDF]

Supplementary Material

ADP-dependent kinases from the archaeal order *Methanosarcinales* adapt to salt by a non-canonical evolutionary conserved strategy

Felipe Gonzalez-Ordenes[1#], Pablo Cea[1#], Nicolás Fuentes[1], Sebastián Muñoz[1], Ricardo Zamora[1], Diego Leonardo[2], Richard C. Garratt[2], Victor Castro-Fernandez[1]* and Victoria Guixé[1]*

[1] Laboratorio de Bioquímica y Biología Molecular, Departamento de Biología, Facultad de Ciencias, Universidad de Chile, Santiago, Chile.

[2] São Carlos Institute of Physics, University of São Paulo, São Carlos, São Paulo, Brazil.

# These authors contributed equally to this work.

*** Correspondence:**

Victor Castro-Fernandez (vcasfe@uchile.cl) and Victoria Guixé (vguixe@uchile.cl)

Supplementary Table S1. Sequences used for homology modeling of ADP-dependent sugar kinases

	Uniprot access code	Organism	Function	NaCl molar range tolerated for growth. (Optimal, M)
1	F7XKY4	*Methanosalsum zhilinae*	PFK/GK-ADP	0.2 - 2.5 (0.6)
2	D7E8P3	*Methanohalobium evestigatum*	PFK/GK-ADP	1.7 – 5.1 (4.3)
3	A0A1L9C332	*Methanohalophilus portucalensis*	PFK/GK-ADP	1 .0 - 3.0 (2.2)
4	D5E7K4	*Methanohalophilus mahii*	PFK/GK-ADP	0.4 - 3.5 (2.0)
5	A0A1L3Q295	*Methanohalophilus halophilus*	PFK/GK-ADP	0.3 -2.6 (1.2-1.5)
6	P58847	*Methanosarcina acetivorans*	PFK/GK-ADP	0.1 – 1.0 (0.2)
7	Q8PZL9	*Methanosarcina mazei*	PFK/GK-ADP	0.1 – 0.8 (0.3)
8	A0A0E3SS17	*Methanococcoides methylutens*	PFK/GK-ADP	0.1 – 1.0 (0.4)
9	L0L1P2	*Methanomethylovorans hollandica*	PFK/GK-ADP	0 – 0.3 (0.04)
10	Q46B04	*Methanosarcina barkeri*	PFK/GK-ADP	0.1 – 0.7 (0.2)
11	A0A0E3L818	*Methanosarcina siciliae*	PFK/GK-ADP	0.2 – 0.8 (0.5)
12	M0CSP0	*Haloterrigena limicola*	PFK-ADP	1.7 – 5.1 (3.1)
13	M0LRE6	*Halobiforma lacisalsi*	PFK-ADP	> 1.7 (2.6 - 4.3)
14	L9XHX4	*Natronococcus Jeotgali*	PFK-ADP	1.3 – 5.1 (4.1)
15	L9WMF7	*Natronorubrum bangense*	PFK-ADP	2.1 – 4.3 (3.9)
16	L9XEP3	*Natronococcus amylolyticus*	PFK-ADP	1.4 – 5.2 (3.1)
17	L0IBN8	*Halovivax ruber*	PFK-ADP	> 2.5 (3.4)
18	Q8VDL4	*Mus musculus*	GK-ADP	D.A
19	F7DTI9	*Xenopus tropicalis*	GK-ADP	D.A
20	A0JML7	*Danio rerio*	GK-ADP	D.A
21	Q9VUB9	*Drosophila melanogaster*	GK-ADP	D.A
22	Q9BRR6	*Homo sapiens*	GK-ADP	D.A
23	Q86S40	*Caenorhabditis elegans*	GK-ADP	D.A

D. A. Criterion of medium salinity for halotolerance or optimal growth does not apply to these organisms. Halophilic *Methanosarcinales* (light Blue), non-halophilic *Methanosarcinales* (yellow), *Halobateria* (red) and *Eukarya* (green).
