# Supplementary material for: ADP-Dependent Kinases From the Archaeal Order Methanosarcinales Adapt to Salt by a Non-canonical Evolutionarily Conserved Strategy
Source: Front Microbiol. 2018 Jun 26;9:1305. doi: 10.3389/fmicb.2018.01305 (PMC6028617; doi:10.3389/fmicb.2018.01305)
Supplement: Supplementary file 2 [file Table_2.PDF]

Supplementary Material

ADP-dependent kinases from the archaeal order *Methanosarcinales* adapt to salt by a non-canonical evolutionary conserved strategy

Felipe Gonzalez-Ordenes[1#], Pablo Cea[1#], Nicolás Fuentes[1], Sebastián Muñoz[1], Ricardo Zamora[1], Diego Leonardo[2], Richard C. Garratt[2], Victor Castro-Fernandez[1*] and Victoria Guixé[1*]

[1] Laboratorio de Bioquímica y Biología Molecular, Departamento de Biología, Facultad de Ciencias, Universidad de Chile, Santiago, Chile.

[2] São Carlos Institute of Physics, University of São Paulo, São Carlos, São Paulo, Brazil.

# These authors contributed equally to this work.

*** Correspondence:**

Victor Castro-Fernandez (vcasfe@uchile.cl) and Victoria Guixé (vguixe@uchile.cl)

Supplementary Table S2. Outer shell amino acid composition of the homology models analyzed

**Amino acid**	***Eukarya***	***Halobacteria***	**Halophilic *Methanosarcinales***	**Non-halophilic *Methanosarcinales***
**Average Percentage**				
**Ser**	8.85±2.82	5.09±0.68	5.26±0.43	5.11±1.20
**Thr**	4.49±1.34	5.9±0.72	3.19±0.31	3.88±0.63
**Asn**	4.22±0.91	1.43±0.46	6.43±0.60	4.81±0.90
**Gln**	6.12±1.32	2.24±0.17	3.42±0.70	3.35±0.80
**Tyr**	1.72±1.32	3.21±1.32	4.71±0.64	4.3±1.14
**Trp**	1.84±0.57	0.36±0.23	1.16±0.26	1.33±0.26
**Phe**	2.97±0.89	2.7±0.29	2.51±0.54	2.84±0.53
**Met**	1.63±0.68	2.03±0.50	2.2±0.42	1.99±0.59
**Cys**	0.23±0.27	0.41±0.37	1.47±0.79	0.97±0.42
**Pro**	5.38±0.75	6.47±0.66	4.46±0.70	4.38±0.35
**Ile**	3.82±1.04	3.1±0.65	6.73±0.72	4.93±1.57
**Leu**	7.92±1.0	8.34±0.75	6.54±0.62	8.37±1.31
**Val**	5.3±1.4	6.31±0.58	6.05±0.83	5.6±1.86
**Ala**	8.29±2.04	9.78±1.88	3.79±0.57	3.99±0.80
**Gly**	4.83±0.99	5.77±1.36	3.48±1.1	5.07±0.70
**Asp**	5.49±0.57	11.47±1.02	9.48±1.06	7.61±0.97
**Glu**	9.11±1.75	13.28±2.81	10.61±1.56	12.73±2.11
**Lys**	4.89±1.1	1.38±0.44	8.83±2.72	9.44±1.25
**Arg**	8.19±1.15	7.5±1.07	6.43±1.29	6.14±0.80
**His**	4.71±1.63	3.62±0.71	3.25±0.49	3.18±0.51

The numbers represent the average percentage for each amino acid content (n=6 for all groups except for halophilic *Methanosarcinales*, where n=5) +/- s.d.
