# Supplementary material for: ADP-Dependent Kinases From the Archaeal Order Methanosarcinales Adapt to Salt by a Non-canonical Evolutionarily Conserved Strategy
Source: Front Microbiol. 2018 Jun 26;9:1305. doi: 10.3389/fmicb.2018.01305 (PMC6028617; doi:10.3389/fmicb.2018.01305)
Supplement: Supplementary file 3 [file Table_3.PDF]

frontiers

Supplementary Material

ADP-dependent kinases from the archaeal order *Methanosarcinales* adapt to salt by a non-canonical evolutionary conserved strategy

Felipe Gonzalez-Ordenes[1#], Pablo Cea[1#], Nicolás Fuentes[1], Sebastián Muñoz[1], Ricardo Zamora[1], Diego Leonardo[2], Richard C. Garratt[2], Victor Castro-Fernandez[1]* and Victoria Guixé[1]*

[1] Laboratorio de Bioquímica y Biología Molecular, Departamento de Biología, Facultad de Ciencias, Universidad de Chile, Santiago, Chile.

[2] São Carlos Institute of Physics, University of São Paulo, São Carlos, São Paulo, Brazil.

# These authors contributed equally to this work.

*** Correspondence:**

Victor Castro-Fernandez (vcasfe@uchile.cl) and Victoria Guixé (vguixe@uchile.cl)

Supplementary Table S3. Inner shell amino acid composition of the homology models analyzed

**Amino acid**	***Eukarya***	***Halobacteria***	**Halophilic *Methanosarcinales***	**Non-halophilic *Methanosarcinales***
	**Average Percentage**			
**Ser**	7.01±1.48	5.27±1.4	8.11±0.53	8.46±1.03
**Thr**	4.09±0.72	5.59±0.45	2.96±0.52	2.00±1.13
**Asn**	1.56±0.58	3.09±0.95	2.45±0.72	2.28±0.85
**Gln**	1.49±0.87	1.17±0.68	0.98±0.32	1.10±0.52
**Tyr**	2.37±0.97	2.67±0.61	2.70±0.44	3.59±1.64
**Trp**	0.88±0.32	0.27±0.42	0.86±0.34	0.48±0.40
**Phe**	5.72±0.97	2.94±0.61	3.19±0.44	3.51±0.78
**Met**	3.32±0.58	2.64±1.38	1.58±0.91	2.89±0.91
**Cys**	2.14±0.24	1.15±0.66	1.23±0.44	0.81±0.26
**Pro**	3.14±1.15	3.06±0.88	2.59±0.54	2.45±0.51
**Ile**	6.60±1.63	5.18±1.75	15.00±1.56	12.72±1.32
**Leu**	16.63±1.36	12.94±1.91	13.01±1.33	14.81±3.07
**Val**	12.37±2.02	13.64±1.91	12.18±1.26	11.95±1.01
**Ala**	13.08±2.08	22.26±1.41	15.05±2.38	15.15±2.39
**Gly**	9.96±1.52	8.73±1.60	9.84±0.38	9.67±1.27
**Asp**	2.66±1.01	2.54±1.20	1.62±0.94	2.67±0.76
**Glu**	3.01±0.58	2.55±0.91	2.33±1.57	1.26±0.47
**Lys**	0.19±0.30	0.37±0.40	0.61±0.43	0.48±0.55
**Arg**	0.98±1.08	0.78±0.48	1.11±0.28	1.16±0.55
**His**	2.80±1.07	3.17±0.68	2.58±0.80	2.64±0.74

The numbers represent the average percentage for each amino acid content (n=6 for all groups except for halophilic *Methanosarcinales,* where n=5) +/- s.d.
