# Supplementary material for: ADP-Dependent Kinases From the Archaeal Order Methanosarcinales Adapt to Salt by a Non-canonical Evolutionarily Conserved Strategy
Source: Front Microbiol. 2018 Jun 26;9:1305. doi: 10.3389/fmicb.2018.01305 (PMC6028617; doi:10.3389/fmicb.2018.01305)
Supplement: Supplementary file 5 [file Image_2.PDF]

Supplementary Material

ADP-dependent kinases from the archaeal order *Methanosarcinales* adapt to salt by a non-canonical evolutionary conserved strategy

Felipe Gonzalez-Ordenes[1#], Pablo Cea[1#], Nicolás Fuentes[1], Sebastián Muñoz[1], Ricardo Zamora[1], Diego Leonardo[2], Richard C. Garratt[2], Victor Castro-Fernandez[1]* and Victoria Guixé[1]*

[1] Laboratorio de Bioquímica y Biología Molecular, Departamento de Biología, Facultad de Ciencias, Universidad de Chile, Santiago, Chile.

[2] São Carlos Institute of Physics, University of São Paulo, São Carlos, São Paulo, Brazil.

# These authors contributed equally to this work.

*** Correspondence:**

Victor Castro-Fernandez (vcasfe@uchile.cl) and Victoria Guixé (vguixe@uchile.cl)

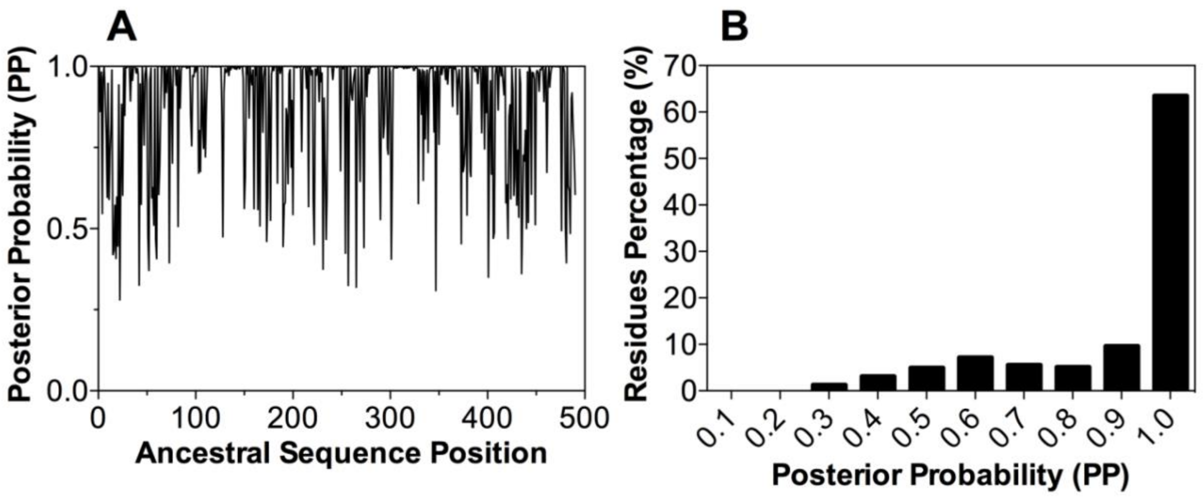

Supplementary Figure S2. Ancestral sequence inference analysis for the AncMsPFK/GK. A. Posterior probability for the ancestral sequence positions. B. Frequency distribution of posterior probabilities (PP).
